# Synthesis, gallium labelling and characterization of P04087, a functionalized phosphatidylserine-binding peptide

**DOI:** 10.1186/s41181-016-0021-5

**Published:** 2017-02-07

**Authors:** Rana Ben Azzouna, Alexandre Guez, Khadija Benali, Faisal Al-Shoukr, Walter Gonzalez, Philippe Karoyan, François Rouzet, Dominique Le Guludec

**Affiliations:** 1 Nuclear Medicine Department and DHU FIRE, Bichat-Claude Bernard University Hospital, AP-HP, Paris, France; 2 UMR 1148 Inserm, Paris, France; 3 Fédération de Recherche en Imagerie Multimodale, Paris 7 University, Paris, France; 4 Sorbonne Universités, UPMC Université Paris 06, Ecole Normale Supérieure, CNRS, Laboratoire des Biomolécules, Paris, France; 5 Guerbet, Research Center, Aulnay-sous-Bois, France

**Keywords:** Phosphatidylserine, Peptide, NODAGA, Gallium, PGDLSR, Infective endocarditis

## Abstract

**Background:**

Radiolabeled phosphatidylserine (PS)-binding peptides represent an innovative strategy for molecular imaging of apoptosis and thrombus. The hexapeptide PGDLSR was described as a selective and high affinity ligand for PS. In this work, we synthesized and evaluated a gallium labelled-PGDLSR peptide as a potential and selective radiopharmaceutical for nuclear imaging. PGDLSR-β-alanine-NODAGA (P04087) was prepared using Fmoc-based synthesis and then chelated with cold gallium, ^68^Ga and ^67^Ga. The affinity of Ga-P04087 for PS was evaluated by a competitive binding assay using biotinylated AnnexinV. The in vitro stability of the radiotracer was checked at room temperature and after incubation in human serum at 37 °C with and without a metalloprotease inhibitor. The in vivo binding of ^67^Ga-P04087 to phosphatidylserine was evaluated in a rat model of infective endocarditis.

**Results:**

PGDLSR was successfully prepared with a yield of 31%. P04087 was obtained with a yield of 28% and in high chemical purity (>95%). The radiochemical purities of ^67^Ga-P04087 and ^68^Ga-P04087 exceeded 98% in all cases. IC50 of P04087 and Ga-P04087 were in the same order of magnitude (10^−7^M). The radiolabelled product was stable for 24 h at room temperature, but was very rapidly degraded in human serum in the absence of a protease inhibitor, which had a stabilizing effect. No focal uptake could be detected visually in the cardiac area on SPECT images. On autoradiography however, a focal uptake of ^67^Ga-P04087 in the valve area was present and histological slices demonstrated localization of peptide binding at the peripheral layer of vegetations.

**Conclusion:**

In spite of the preservation of the peptide affinity to the PS after its conjugation to the NODAGA chelator, and of the presence of ^67^Ga-P04087 uptake on autoradiography, the absence of detectable foci in vivo in the valve area may be attributed to both the low intensity of the signal and the presence of background activity originating from blood pool and surrounding tissues in the living animals. Further modifications are necessary to design a radiolabeled peptide with higher binding potencies to PS while possessing enhanced metabolic stability in vivo.

## Background

The plasma membrane (PM) of a healthy cell generally exhibits an asymmetric distribution of its major phospholipids. Typically, the inner PM leaflet contains essentially all phosphatidylserine (PS), most phosphatidylethanolamine (PE) and phosphatidylinositol (PI), with sphingomyelin (SM) largely confined to the outer leaflet, and phosphatidylcholine (PC) distributed equally between both leaflets. (van Meer 1993); (Martin et al. 1995).

Apoptosis induces redistribution of membrane phospholipids of the cell. In fact, apoptosis, is accompanied by changes in PS distribution at the PM (Martin et al. 1995). PS, being usually constitutive of inner leaflet of the membrane, is then exposed on the outer leaflet. PS exposure is widely considered to be an early marker of apoptotic cell death. (Galluzzi et al. 2012), (Kroemer et al. 2009).

PS externalization precedes several other events (such as loss of membrane integrity…) normally associated with this mode of cell death (Martin et al. 1995). This phenomenon allows the recognition of apoptotic cells by macrophages and their phagocytosis.

Furthermore, in erythrocytes and platelets, PS is normally restricted to the inner leaflet of the bilayer. When platelets are activated or erythrocytes are loaded with Ca^2+^, PS transversely moves across the bilayer to the outer leaflet of the plasma membrane, generating an active surface for assembly of the coagulation factors Va and Xa into the prothrombinase complex (Verhoven et al. 1995). PS exposure occurs also in T-cell activation, without cell death (Kroemer et al. 2009).

PS is accessible to a potential ligand only during the apoptotic process, platelet or T-cell activations (Rouzet et al. 2009), providing an abundant molecular target for the detection of cellular injury.

Several ligands were developed as molecular imaging agents of PS. Annexin V, the endogenous ligand of PS (Andree et al. 1990), was successfully evaluated as a molecular imaging agent of apoptotic cells (Blankenberg et al. 1998) as well as thrombus (Sarda-Mantel et al. 2006), with many potential clinical applications in oncology, cardiovascular diseases, and rheumatology (Boersma et al. 2005).

Annexin V and all protein-ligands (such as synaptotagamin I) that bind to phospholipids in cell membranes (Jung et al. 2004) have however many drawbacks, i.e. the cost of production, their antigenicity, and the restrained diffusion to the targeted sites due to their larger size. (Burtea et al. 2009). Moreover, the high liver and kidney uptake levels of Annexin V lead to low target-to-background ratios (Wuest et al. 2015).

In order to overcome these disadvantages, peptide based approaches represent a promising alternative. Indeed, peptides mimicking protein secondary structures, can bind to proteins with high affinity. As a result, they can serve as agonists, antagonists and allosterics modulators for many receptors and they also have a lower toxicity than small molecules. (Craik et al. 2013), (Bhat et al. 2015), (Tsomaia 2015). Their major drawbacks remain the poor overall properties regarding metabolic stability, oral bioavailability, membrane permeability and conformational stability, limiting their selectivity with the target.

Nevertheless, they have an affordable synthesis and can incorporate a wide variety of functional groups. A new generation of therapeutics peptides has emerged, increasing their conformational stability and their pharmacokinetics properties. (Wójcik and Berlicki 2016).

With this aim in mind and as a valuable alternative to protein-ligands, Burtea et al. provided PS-specific peptides identified by phage display technology that bind targets with high affinity and specificity: R824 (PGDLSR, IC_50_ = 1.33 × 10^−8^M), R825 (DAHSFS, IC_50_ = 8.16 × 10^−7^M) and R826 (LIKKPF, IC_50_ = 1.48 × 10^−8^M) (Burtea et al. 2009).

Thus, if labelled with a suitable radioisotope, these peptides might provide a convenient imaging tool. The ^68^Ga has a very favourable positron emission (1880 keV, 90%) for PET imaging, and a short half-life (68 min) suitably matched for imaging with peptide vectors. (Price and Orvig 2014). The availability of ^68^Ga from a ^68^Ge/^68^Ga generator (^68^Ge t_1/2_ = 270 days) allows hospitals low-cost access to a PET isotope without expensive cyclotron facilities (Ma et al. 2016), (Velikyan 2013).

Herein, after the Fmoc synthesis, the stability study of the three peptides and the selection of the most suitable one, we describe the conjugation of the selected peptide with a metal chelator, the characterization of the functionalized peptide and its radiolabelling with ^68^Ga and with ^67^Ga, a longer-lived isotope of gallium which can be a more suitable tool for preclinical investigations when developing a new Ga-radiotracer.

## Methods

### Synthesis of R824 (PGDLSR), R825 (DAHSFS) and R826 (LIKKPF)

#### Loading of the first amino acid on 2-chlorotrityl resin

Fmoc-protected amino acids were obtained from commercial suppliers (Senn Chemicals, Iris Biotech GmbH, Fluka, and Novabiochem). The first Fmoc-protected amino acid was loaded on the 2-Cl-trityl resin (Iris Biotech GmbH).

A solution of the Fmoc-Amino Acids (0.3-1.2 eq. relative to the resin, depending on the final loading expected) and Diisopropylethylamine (DIPEA) (4 eq. relative to the Fmoc-amino acid) in dichloromethane (DCM) (10 mL per gram of resin) was added to the DCM-swollen resin. The suspension was shaken at room temperature for 2 h, then washed with a mixture of DCM, methanol (MeOH) and DIPEA (17:2:1, 3×), DCM (3×), dimethylformamide (DMF) (3×), MeOH (3×), and dried in vacuo.

Synthesis was performed on a 0.25 mmol scale.

#### Peptide coupling

The resin-linked peptide was swollen with N-methyl-2-pyrrolidone (NMP) and then was treated with a solution containing the Fmoc-protected amino acid (5 eq.), O-(Benzotriazol-1-yl)-N,N,N’,N’-tetramethyluronium hexafluorophosphate (HBTU) (5 eq.), N-hydroxybenzotriazole (HOBt) (5 eq.) and DIPEA (10 eq.) in NMP (10 mL per gram of resin). The suspension was shaken for a duration extending from 30 min to a whole night depending on the amino acid that was used.

For removal of the N-terminal Fmoc groups, the resin was first treated with a 20% solution of piperidine in NMP for 3 min and subsequently with a 20% solution of piperidine in NMP for 15 min. Finally, the resin was washed with NMP.

The completion of the reaction is monitored by a Kaiser test (Kaiser et al. 1970).

#### Simultaneous cleavage and deprotection of peptide

The resin was treated with a mixture of trifluoroacetic acid (TFA)-H_2_O- triisopropylsilane (TIS), 95:2.5:2.5 and shaken at room temperature for 2 h. After removal of the volatile compounds under reduced pressure, the fully deprotected peptide was precipitated by addition of pre-cooled diethyl ether (Et_2_O) and centrifuged for 5 min at 6,000 rpm. The ether was decanted and the same operation was repeated twice. The crude product was lyophilized and finally purified by preparative RP-HPLC using a C18 column. Purity was determined by analytical RP-HPLC on linear gradient 5 to 35% of CH_3_CN (0.1% TFA) in H_2_O (0.1% TFA) in 30 min.

#### Quality control of the prepared peptides

Peptides were characterized by MALDI-TOF Mass Spectrometry (DE-Pro, PerSeptive Biosystems) in positive ion reflector mode using the matrix α-Cyano-4-hydroxycinnamic acid. The m/z of the protonated molecule (first isotope) are given as theoretical (calculated), experimental (found).

### Selection of the peptide-candidate to be developed as a nuclear contrast agent

A 100 μL solutions of R824 (PGDLSR), R825 (DAHSFS) and R826 (LIKKPF) in Hank’s medium and 10% FCS (foetal calf serum) were prepared and incubated (in triplicate) for 2 h at 37 °C and in complete darkness. At 0, 1 h and 2 h, 100 μL were sampled from each tube; 200 μL of 0.6 M trichloroacetic acid were added. The mixtures were then centrifuged at 20,000 g for 10 min at 4 °C. One microliter of each supernatant was then analyzed by LC/ES/MS.

### Synthesis of P04087

R824-β-alanine was firstly prepared as described above.

**Fig. 1:**
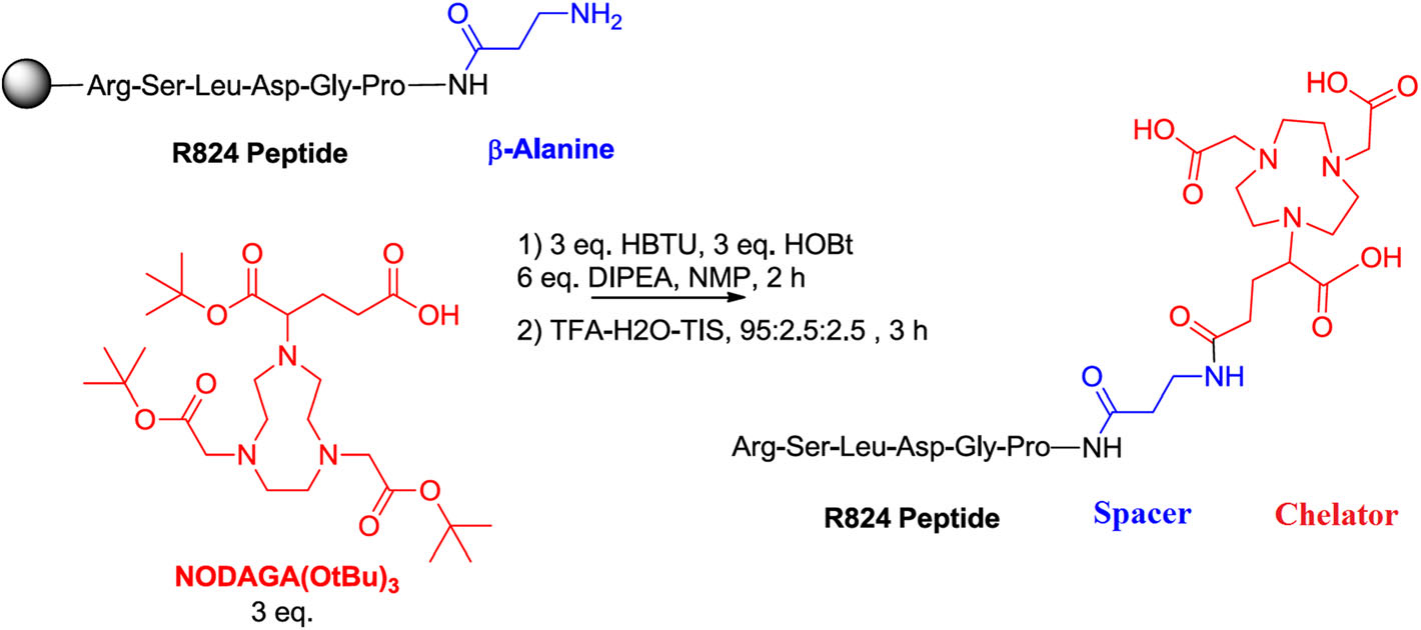
The coupling of R824-β-Alanine and NODAGA

The NODAGA chelator was conjugated directly to the peptide R824 resin bound via a spacer (beta-alanine). Coupling of NODAGA was performed with only 3 equivalents of HBTU, HOBt and 6 equivalents of DIPEA as detailed in Fig. 1.

### Synthesis of cold gallium-P04087

**Fig. 2:**
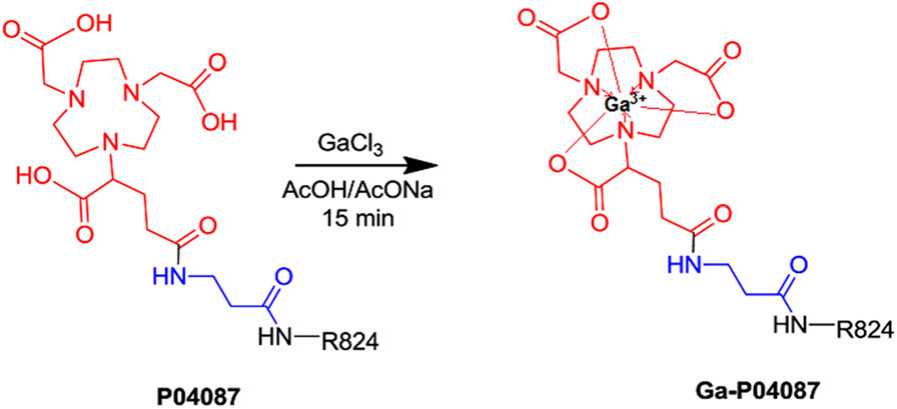
Labelling of P04087 with natural gallium

P04087 was chelated with cold gallium as described in Fig. 2. Gallium (III) chloride (^nat^Ga) was purchased from Sigma-Aldrich.

P04087 peptide (18 mg, 14 μM) in 2 mL of an acetic acid and sodium acetate buffer (pH = 5.0) was incubated with 31 μL of a gallium (III) chloride 0.5 M solution (1.1 eq.), for 15 min at room temperature. Following this incubation period, the sample was directly injected into a q-TOF mass spectrometer.

The quantification of the labeling was determined by calculating the relative peak height of the molecular masses corresponding to the chelated and unchelated peptide.

The purification unit consisted of a Sep-Pak C18 cartridge (Waters, 1 g C18). The C18 was conditioned with 1 mL of absolute ethanol followed by 3 mL deionized water. The reaction mixture was transferred to the C18 cartridge. After adsorption, the cartridge was washed by 2.5 mL of deionized water (in order to wash out residual unreacted GaCl_3_) and then, the purified product was eluted by 2 mL of a mixture acetonitrile/water (1:1). The resulting solution was then dried on a rotary evaporator, mixed with 5 mL of H2O, frozen at −78 °C and subsequently lyophilized overnight.

### Affinity study

Affinities of cold-labelled peptide (Ga-P04087) and the peptide of reference (R824) for phosphatidylserine were evaluated by enzyme-linked immunosorbent assay. A competitive binding assay procedure was performed: A 96-well plate (MICROLON plaque 200, med. Binding, F-bottom, GREINER) was coated with 200 μL/well of phosphatidylserine (PS) (Avanti Polar Lipids) (dissolved in ethanol (SIGMA ALDRICH) for a final concentration of 100 μg/mL). The lipids were dried overnight at room temperature under a ventilation hood, and the plate was washed three times with Ca^2+^-Tween-20 0,05 buffer (10 mM HEPES (pH 7.4), 150 mM NaCl, 2.5 mM CaCl_2_, 0.05% Tween-20) to remove any nonbinding lipids. Next, Blocking Solution (300 μL) (Protein-Free (TBS) and Blocking Buffer from Thermo Fisher Scientific) were added to each well, and the plates were incubated at room temperature for 1 h on a shaker to block nonspecific binding. Wells were washed with Washing Buffer for 5 min (this being done twice). Serial dilutions of peptides (from 0.01 nM to 1 μM) in Ca^2+^ Buffer (10 mM HEPES, 150 mM NaCl, 2.5 mM CaCl_2_, pH 7.4) were incubated at 37 °C for 30 min with PS immobilized on microtiter plates. The competitor (biotinylated AnnV: Annexin V-Biotin Reagent from CLINISCIENCES) was then added at a concentration equal to its Kd for PS (5 × 10^−10^ M) and incubated for 1 h 30. After rinsing with Ca^2+^ buffer, the binding between AnnV-Bt and the target was detected by adding the ABC Standard Peroxidase Staining Kit (Thermo Fisher Scientific) (200 μL) for more than (over) one hour at room temperature. The staining reaction was developed with 200 μL/well of ABTS (22 mg ABTS (SIGMA ALDRICH) in 100 mL of 50 mM sodium citrate, pH 4.0 completed with 0.05% H_2_O_2_ (SIGMA ALDRICH). OD405 values were measured using a microplate reader StatFax-2100.

### Radiolabelling with gallium 68

The ^68^Ga was available from a chemical grade ^68^Ge/^68^Ga generator system (Cyclotron Co., Ltd, Obninsk, Russia) where ^68^Ge was attached to a column of an inorganic matrix based on titanium dioxide. The pre-treatment of the chemical grade eluate is necessary before the labelling operation. Thus, a synthesizer is needed.

An automated radiolabelling of P04087 with ^68^Ga was performed with Fastlab synthesizer (GE Healthcare) using an approach combining the fractioned elution and anionic purification eluate with a Chromafix 30-PS-HCO3 resin (Macharey-Nagel), which was validated by Velikyan et al. (Velikyan et al. 2004).

The generator was eluted by 0.1 M HCl solution (sigma-aldrish). The middle fraction was collected and mixed with high concentrated HCl solution (30%, Merck). The obtained gallium chloride was then loaded in the anion exchange resin. The elution of the purified gallium was performed with ultrapure water (500 μL) to reactor which contains 65 μL of a 1 mg/mL P04087 solution in ultrapure water (TraceSELECT, Fluka) mixed with 0.4 M sodium acetate Eur. Phar. buffer, pH 4.6.

The preparation was incubated for 5 min at room temperature, was diluted with saline and then analysed by radio-HPLC without any further purification.

The radiochemical purity was determined by HPLC system from Dionex (DIONEX UltiMate 3000) consisting of HPLC pump, quaternary gradient unit, LPG-3400 SD DIONEX; autosampler, WPS-3000 SL DIONEX; multiwavelength detector, DAD-3000 DIONEX; column oven, TCC-3000 DIONEX and a radio detector, HERM LB 500 Berthold, Germany, coupled in series. Data acquisition and handling was performed using Chromeleon software, DIONEX.

The following HPLC conditions were used: ACE® column 100 Å C18 with the dimensions 150 mm × 4.6 mm, 3 μm particle size. We applied gradient elution with the following parameters: A) H2O, 0.1% TFA; B) acetonitrile, 0.1% TFA; with UV detection at 220 nm; flow was 1 mL/min; 0–2 min isocratic 5% B, 5-35% B linear gradient 20 min, 35-5% B linear gradient 2 min. Retention time (tR) for the radio-HPLC signal was 11.7 ± 0.07 min.

### Radiolabelling with gallium 67

Radiolabelling with a longer-living isotope of gallium (i.e. (the) gallium 67) is more convenient for the preclinical investigations in parallel to the design of a new ^68^Ga-vector.

#### Preparation of [^67^Ga]-chloride from [^67^Ga]-Citrate

Gallium 67 chloride was prepared from citrate solution (iba molecular) following the method described by Vladimir Scasnar and Johan E. van Lier. The gallium citrate solution was mixed with two thirds of its volume of ultrapure water (TraceSELECT®, Fluka Analytical. Sigma-Aldrich-Switzerland. The solution was then filtered manually over a 100 mg Sep-Pak® Vac 1 cc (100 mg) silica cartridges (Waters, Ireland) with a plastic syringe. The cartridge was washed twice with 5 mL ultrapure water to remove citrate ions. The retained radioactivity was eluted from the cartridge with 1 mL of 0.1 M hydrochloric acid solution (BioXtra, Sigma-Aldrich, Germany) to yield a ^67^GaCl_3_ solution free from citrate ions that can be used for radiolabelling. (Scasnár and van Lier 1993).

The radiochemical purity of the gallium chloride solution was tested by radio-thin layer chromatography using ITLC-SG (PALL), mobile phase: (0.4 M sodium acetate pH 4 which was prepared using sodium acetate trihydrate BioUltra ≥ 99.5%, sigma-aldrich, Germany, Water TraceSELECT®, Fluka Analytical. Sigma-Aldrich – Switzerland. pH was adjusted by addition of acetic acid ≥99.99%, Sigma-Aldrich).

Retention was expressed as % of trapped ^67^Ga on the silica cartridge from the total ^67^Ga-Citrate activity passed through the column. Desorption was expressed as % eluted ^67^Ga of total ^67^Ga activity trapped on the column.

The recovery yield was expressed as % of eluted ^67^Ga from the total ^67^Ga-citrate solution activity passed through the silica cartridge.

^67^Ga activity was counted with an ionization chamber (scintidose, LemerPax).

#### Radiolabelling of P04087 with gallium 67

Sixty five μL of 1 mg/mL P04087 solution in 0.4 M sodium acetate pH 4.6 were mixed with 500 μL of the same buffer (Eur. Pharmacopeia buffer) and the ^67^GaCl_3_ solution, prepared as described above (activities from 143 to 264 MBq). The preparation was incubated for 5 min at room temperature.

The End of Synthesis (EOS) activity was measured by means of a calibrated radionuclide activity meter (Scintidose, LemerPax).

#### Stability of the gallium radiotracer

The stability of the radiolabelled peptide was checked at room temperature first 4 h and then 24 h after the end of radiolabelling by HPLC using the gradient described above.

The metabolic stability was checked in human serum at 37 °C in presence and in absence of 1,10 phenanthroline monohydrate, a well-known metalloprotease inhibitor (Wijayanti et al. 2006) at appropriate periods of time (0, 15, 30, 60 min).

In two different tubes containing 200 μL of human serum plasma previously incubated at 37 °C was added 40 μL of ^67^Ga-P04087 labelling solution. 4 μL of 1 mg/mL of 1,10 phenanthroline monohydrate in ethanol was added only in one of the two tubes.

At 0, 15, 30 and 60 min, 20 μL aliquots were collected and proteins were precipitated with 200 μL of methanol. Samples were then filtered with 0.2 μm Whatman filter (13 mm) and the methanol was then evaporated with a stream of nitrogen.

The resulting dry residue was dissolved in 200 μL water and then analyzed by C18-radio-HPLC using the equipment mentioned above.

#### The in vivo binding to phosphatidylserine

We evaluated the in vivo binding of ^67^Ga-P04087 to PS in a rat model of infective endocarditis. This model was selected because we previously evidenced the high expression of PS in valve vegetations in relation with platelets activation (Rouzet et al. 2008). The procedures and animal care complied with the principles of animal care formulated by the National Society for Medical Research. This study was conducted under authorization of the French Direction of the Veterinary Services (No. 75–214) and the approval of the Animal Ethics Committee of our Institution. Infective endocarditis was induced in three males Wistar rats (Janvier, France) as described previously (Rouzet et al. 2008). In brief, a polyethylene catheter was inserted into the left ventricle through the right carotid artery in anesthetized rats (ketamine/xylazine). The catheter remained indwelling throughout the experiment to induce an aseptic thrombotic vegetation formation on aortic valves. Twenty-four hours after catheterization, rats underwent bacterial inoculation (10^8^ colony forming units of Enterococcus faecalis JH2-2) by the left jugular vein under halothane anesthesia. Imaging was performed 4 days after bacterial inoculation. Animals were injected with 60 MBq of ^67^Ga-P04087, and underwent whole body SPECT/CT 1 h later (NanoSPECT/CT plus; Bioscan Inc.). Reconstructed images were assessed visually to determine the presence of a focal tracer uptake in the heart area and were quantified using the ratio between myocardium to lung activity (uptake ratio).

After completion of imaging procedure, animals were euthanized with pentobarbital overdose. Tissue samples were frozen and cut into 20 μm thickness sections, which were exposed in a digital beta imager (BetaIMAGER, Biospace Lab, Paris, France) for 8 h. For histologic examination, tissue samples were fixed in buffered paraformaldehyde (4% v/v, for 48 h) to obtain better morphologic definition, embedded in paraffin and cut into 5 μm thick slices. Sections were stained with hematoxylin-eosin for morphologic analysis of cells and nuclei and with Alcian blue coupled with nuclear red to reveal areas of mucoid accumulation and their relation to cell.

## Results

### Synthesis of R824, R825 and R826 peptides

R824, R825 and R826 peptides were obtained as a white powder with a yield of 29-32% and in high chemical purity (>95%). Molecular masses were verified by mass spectrometry (Table 1).

**Table 1:** MS analysis of R824, R825 and R826

Peptide	Sequence	Molecular mass		Purity (%)	Yield (%)
		Calculated	Found		
R826	LIKKPF	745.5	745.5	97	32
R825	DAHSFS	662.4	662.3	98	29
R824	PGDLSR	644.3	644.3	98	31

### Stability study of R824, R825 and R826

The stability results of the three peptides are shown in Table 2

**Table 2:** Stability study of R824, R825 and R826

Peptide	Sequence	Media	Temperature (°C)	Time of incubation (h)	Stability result
R826	LIKKPF	Hanks + 10% FCS	37	2	Total degradation 1 h after the start of incubation
R825	DAHSFS	Hanks + 10% FCS	37	2	Stable
R824	PGDLSR	Hanks + 10% FCS	37	2	Stable

**Table 3:** MS analysis of P04087 peptide

Compound	Peptide sequence	Molecular mass	
		Calculated	Found
P04087	NODAGA-βAla-PGDLSR	1072.5	1072.4

.

While the R826 peptide is quickly degraded (1 h) in Hanks media supplied or not with foetal calf serum (FSC), R824 and R825 appear stable for two hours in the same conditions at a temperature of 37 °C. The R824 hexapeptide was selected for further investigations in the design of a contrast agent suitable for nuclear imaging, because of its higher PS affinity compared with R825 (Burtea et al. 2009).

### Synthesis of R824-β-alanine-NODAGA (P04087)

R824 peptide (PGDLSR) was successfully prepared using the Fmoc-based synthesis (31% yield). The NODAGA chelator was conjugated to the peptide R824 via a spacer (beta-alanine) at the N-terminus. After purification, the NODAGA conjugated peptide (P04087) was obtained as a white powder with a yield of 28% and in high chemical purity (>95%).

Molecular masses were verified by mass spectrometry (Table 3).

### Cold labeling of P04087 peptide with ^nat^Gallium (III) chloride

Cold gallium labelling was successfully performed to provide Ga-P04087.

**Fig. 3:**
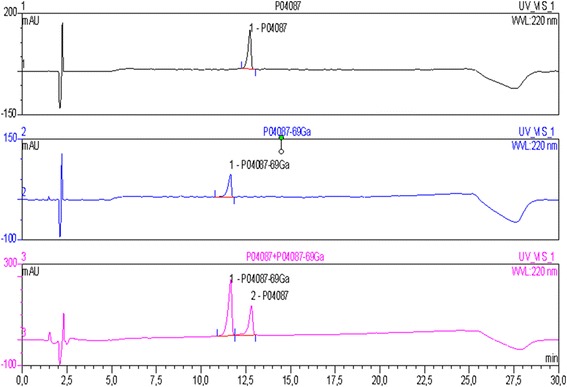
HPLC chromatograms of (**1**) P04087 (RT = 12.7), (**2**) Ga-P04087 (RT = 11.7) and (**3**) a mixture (P04087/Ga-P04087)

HPLC chromatograms of P04087, Ga-P04087 and a mixture of the two compounds are given in Fig. 3.

### Affinity study

**Fig. 4:**
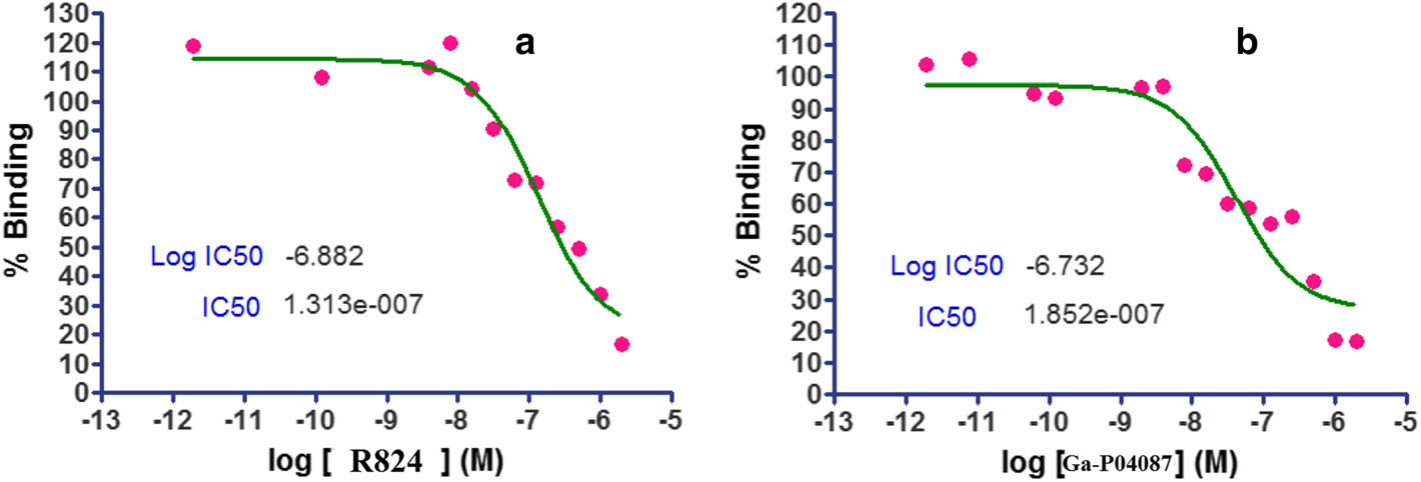
Phosphatidylserine saturation by (**a**) R824 peptide, (**b**) the Ga-P04087 tracer. When Annexin V is present without competitor, the signal is considered as reference and is equal 100%

The affinities of (Ga-NODAGA-ßAla-R824) and R824 were evaluated by ELISA. (Fig. 4). The annexin V concentration was stable. Concentrations of tested compounds were increased from 0.01 nM to 1 μM. The IC_50_ of R824 (1.313x10^−7^M)) and Ga-P04087 (1.852 x 10^−7^M) were in the same order of magnitude. Thus, the chemical modifications (the addition of a spacer, a chelator and Ga) did not alter the affinity of R824 to its target, i.e. the PS.

### Radiolabelling

#### The preparation of [67] gallium chloride from [^67^Ga]-Citrate

**Fig. 5:**
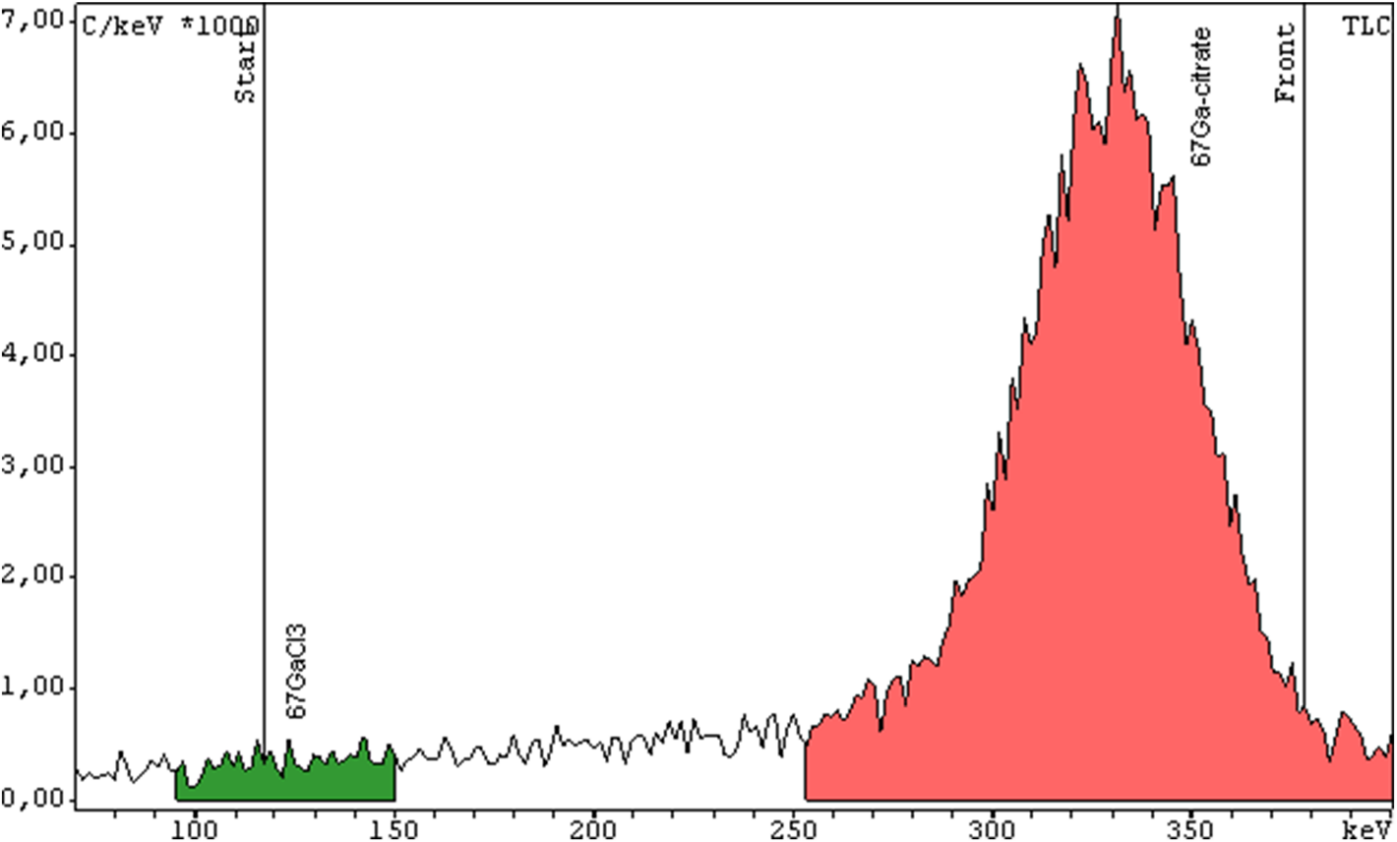
Radio-ITLC-SG of the ^67^Ga-citrate solution purchased from iba molecular. Mobile phase: 0.4 M sodium acetate pH 4. Rf (67Ga-citrate) = 1

Five preparations of gallium chloride were performed. Prior to each preparation, radiochemical purity of gallium citrate solution was controlled with ITLC-SG using 0.4 M sodium acetate (pH 4) as mobile phase. A typical radiochromatogram is shown in Fig. 5.

The volume of citrate solution that passed through the silica cartridge ranged from 0.84 to 3 mL and activities were from 131 to 517 MBq. The details of the experiments are shown in Table 4.

**Table 4:** Details of preparation of gallium chloride solution from gallium citrate solution

^67^Ga-citrate volume (mL)	67Ga-Citrate activity (MBq)	67Ga activity trapped in silica cartridge (MBq)	Retention yield (%)	Eluted activity (MBq)	Desorption yield (%)	^67^GaCl_3_ recovery yield (%)	RCP (%)
0.9	150.8	134.8	89.39	131.65	97.66	87.3	99.6
0.84	124	104.9	84.60	102.3	97.52	82.5	99.83
1	173.2	151.6	87.53	148.6	98.02	85.8	98.38
1	159.6	142.9	89.54	141.46	98.99	88.63	99.99
3	503.24	475.5	94.49	468.7	98.57	93.14	99.89
		Average ± SD	89.11 ± 3.61		98.15 ± 0.62	87.47 ± 3.90	99.54 ± 0.66

**Fig. 6:**
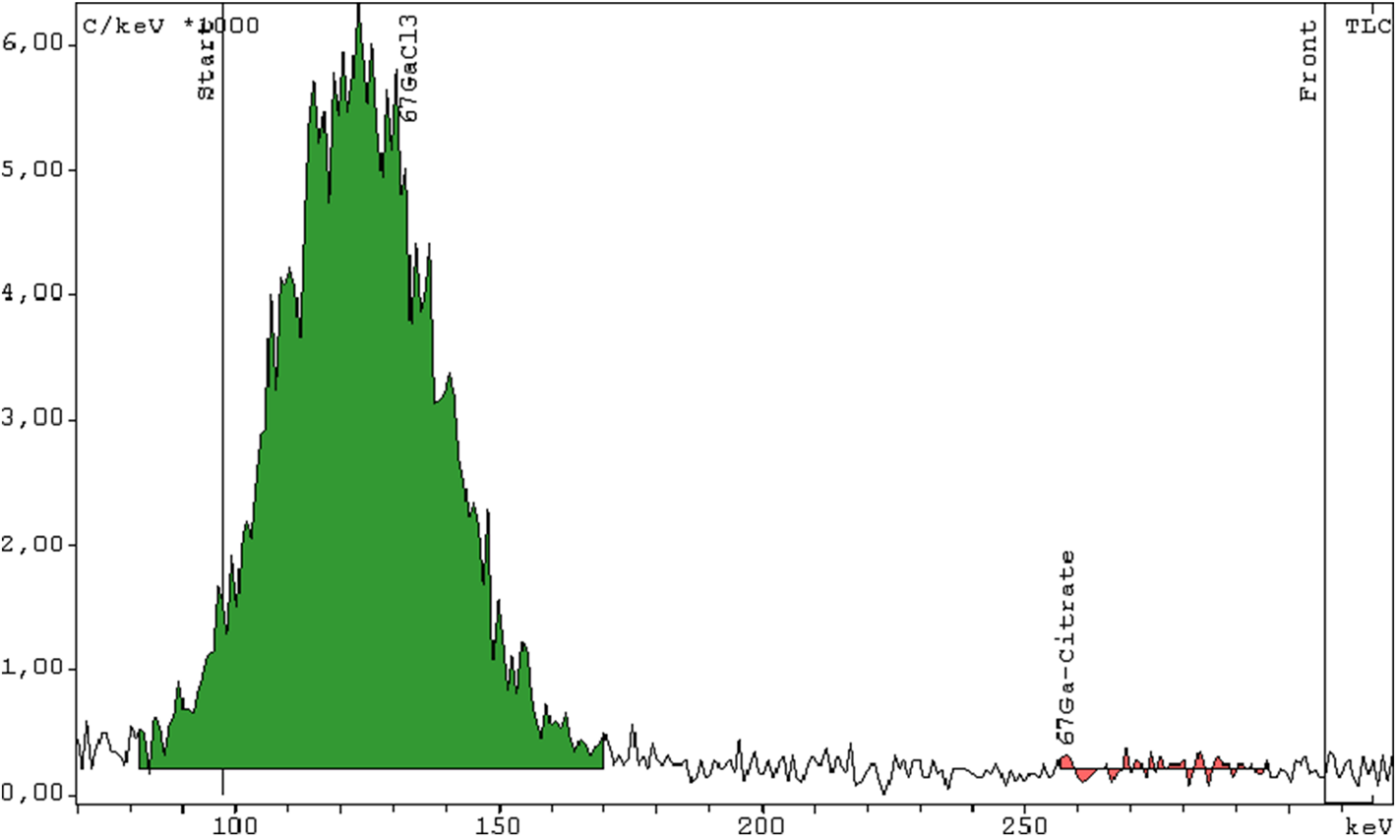
ITLC-SG of the ^67^Ga-chloride obtained by the elution of ^67^Ga from Sep-Pak SI cartridge using 0.1 M HCl. Mobile phase: 0.4 M sodium acetate pH 4. Rf (^67^GaCl3) = 0, Rf (^67^Ga-citrate) = 1 The same retention profile was obtained when ^68^GaCl^3^ eluate was analyzed using the same ITLC-SC conditions

Radiochemical purity, as determined by instant thin-layer chromatography (Fig. 6), exceeded 99% in all cases; the obtained gallium chloride solutions can be used for radiolabelling.

#### The radiolabelling of P04087

The results of the radiolabelling of P04087 with gallium 68 are shown in Table 5.

**Table 5:** Average results of three productions of ^68^Ga-P04087 conjugated peptide

Parameter	Test method	Average ± SD
Radiochemical purity (%)	Radio-HPLC	99.53 ± 0.81
pH	pH meter	4.37 ± 0.32

The results of the radiolabelling of P04087 with gallium 67 are shown in Table 6.

**Table 6:** Average results of three productions of ^67^Ga-P04087 conjugated peptide

Parameter	Test method	Average ± SD
Radiochemical purity (%)	Radio-HPLC	98.84 ± 1.22
pH	pH paper	4.5 ± 0

**Fig. 7:**
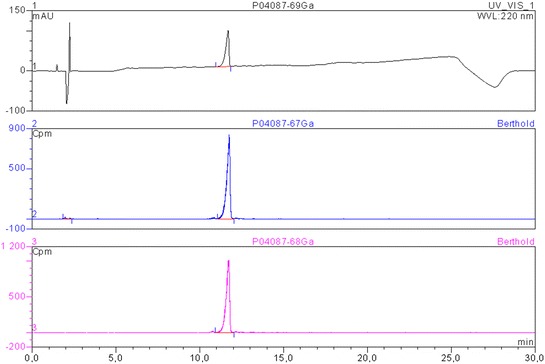
Typical HPLC chromatogram of (*1*) ^Nat^Ga-P04087 and radio-chromatograms of (*2*) ^67^Ga-P04087 and (*3*) ^68^Ga-P04087. (Analytic conditions: column : ACE® 100 Å C18; 150 mm × 4.6 mm, 3 μm particle size. Gradient elution: A) H2O, 0.1% TFA; B) acetonitrile, 0.1% TFA; UV detection at 220 nm; flow: 1 mL/min; 0-2 min isocratic 5% B, 5-35% B linear gradient 20 min, 35-5% B linear gradient 2 min. Retention time (tR) for signal was 11.7 ± 0.07 min

The radiochemical purities were checked by radio-HPLC. Typical radio-chromatograms are shown in Fig. 7.

#### The stability of ^67^Ga-radiotracer

The radiochemical stability of the final radiolabelled product:

The radiochemical purity was checked at room temperature 24 h after the end of the labeling and was 99.60 ± 0.35%. No radiolysis breakdown products were observed, thus highlighting the stability of the final radiolabelled ^67^Ga-radiotracer.

#### The stability in human serum

**Fig. 8:**
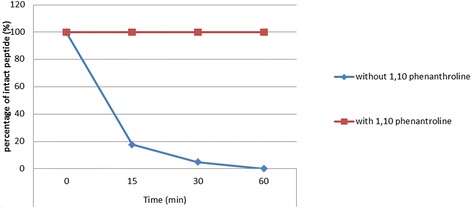
Degradation kinetics of [Ga]-P04087 in human serum. Gallium radiolabelled P04087 peptide was incubated in human serum at 37 °C in presence and in absence of a metalloprotease inhibitor

The in vitro stability of the radiolabelled peptide was evaluated in human serum at 37 °C. As shown in Fig. 8, without metalloprotease inhibitor, the radiolabelled peptide was quickly degraded. In contrast to that rapid degradation, the addition of a suitable amount of 1.10 phenanthroline monohydrate had a stabilizing effect (the 100% radiochemical purity remained stable throughout the study).

#### The in vivo binding to phosphatidylserine

**Fig. 9:**
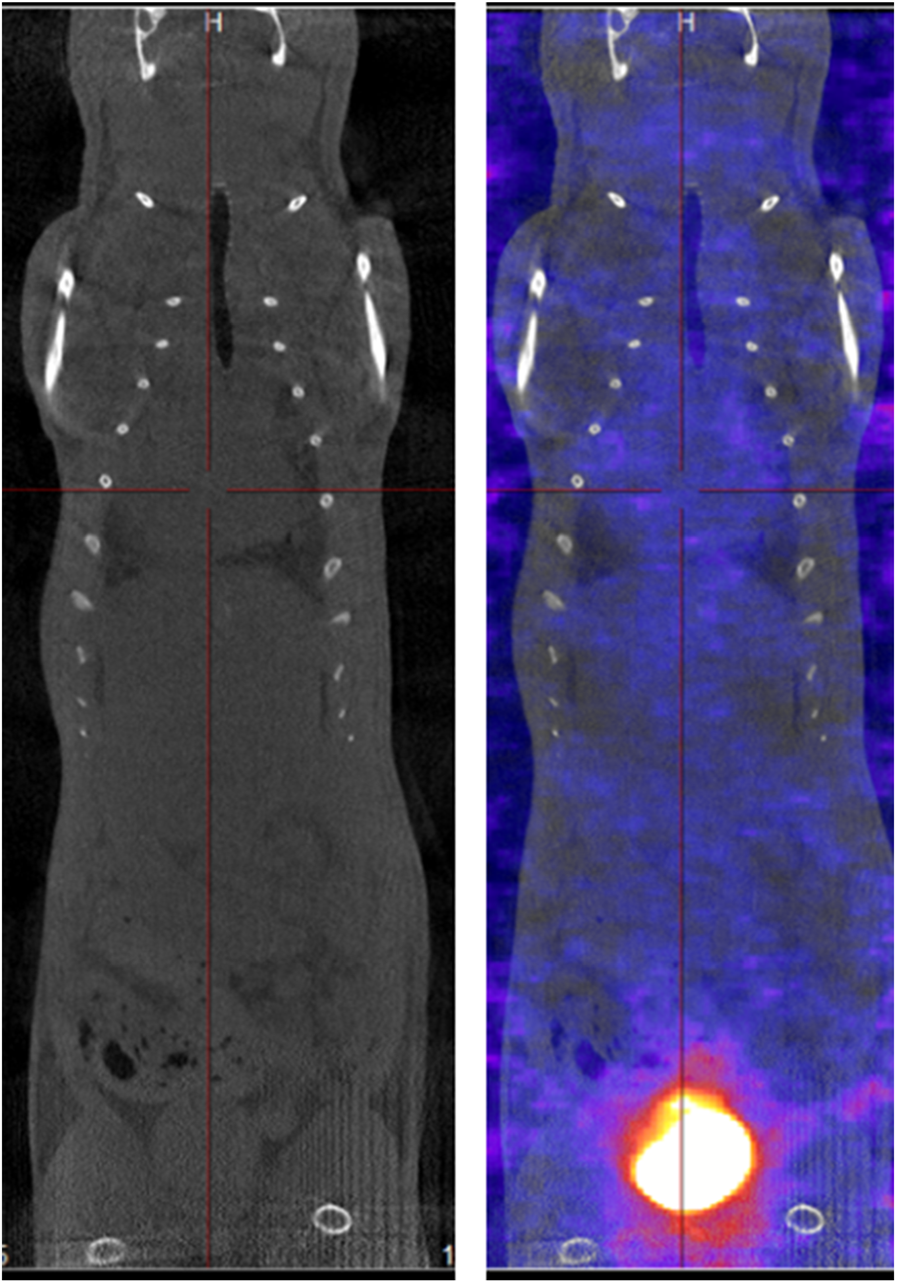
Infective endocarditis in rat. Whole body SPECT/CT acquired 10 minutes after intravenous injection of 67Ga-P04087 showing intense signal in the bladder due to rapid renal elimination. There is no detectable uptake in the heart area

**Fig. 10:**
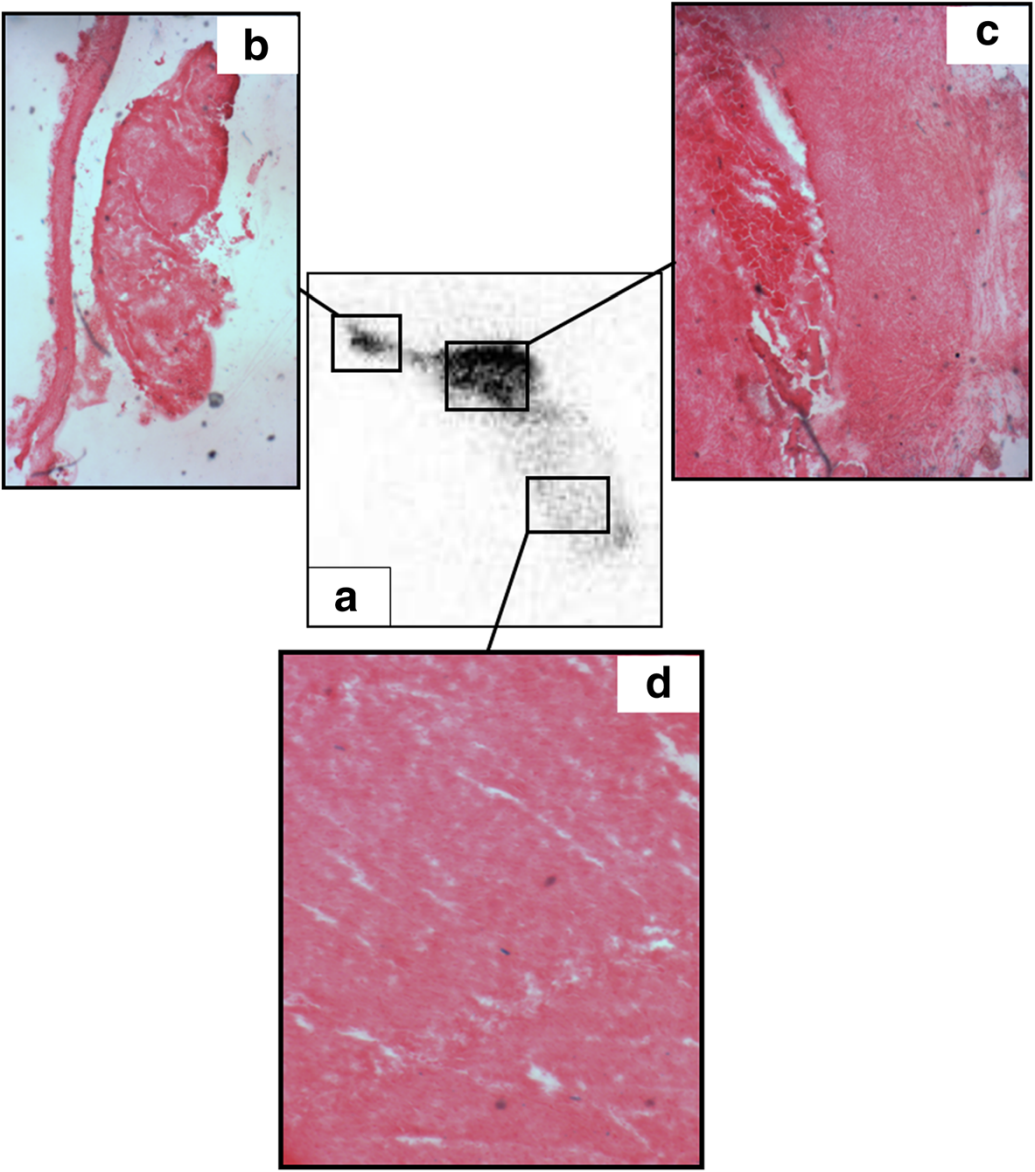
Infective endocarditis in rat. Comparative analysis of autoradiography (**a**) and histology (hematoxylin-eosin staining, ×10) of adjacent slices showing localization of 67Ga-P04087 uptake matching with vegetations on aorta root (**b**) and aorta valve (**c**), and the absence of uptake in remote myocardium (**d**)

No focal uptake could be detected visually in the cardiac area on SPECT images (Fig. 9). On autoradiography however, a focal uptake of ^67^Ga-P04087 in the valve area was present (mean vegetation to remote myocardium ratio: 3.2). Comparative analysis of autoradiography and histological slices demonstrated localization of peptide binding (whatever the tracer) at the peripheral layer of vegetations, corresponding to the recruitment of activated platelets from the blood pool (Fig. 10: Comparative analysis of autoradiography (a) and histology (hematoxylin-eosin staining, ×10) of adjacent slices showing localization of ^67^Ga-P04087 uptake matching with vegetations on aorta root (b) and aorta valve (c), and the absence of uptake in remote myocardium (d).

## Discussion

In this study we described the design of a peptide-based radiotracer labelled with Gallium isotopes that might represent a promising alternative to protein-based radiotracers, regarding their low toxicity, improved stability, low immunogenicity, affordable synthesis compared to protein production which is expensive (Burtea et al. 2009) and easy incorporation of a wide variety of functional groups. As drug candidate, peptides have also higher activity per mass than proteins (Burtea et al. 2009).

With this aim, the Fmoc synthesis of PGDLSR, its conjugation with a metal chelator (NODAGA), the evaluation of its affinity to the phosphatidylserine as a biomarker of apoptotic process and platelet activation, its radiolabelling with ^68^Ga and with ^67^Ga, the evaluation of its stability in human serum and the evaluation of the in vivo binding to PS were reported.

The use of Fmoc-based synthesis and the conjugation approach described here led to a conjugated peptide with high chemical purity and which can be labelled with gallium isotopes in high radiochemical purity.

The affinity studies of our labelled conjugated peptide showed that the adding of a spacer and of a chelating group in the Peptide N-terminus side did not alter the affinity and binding of R824 to its target. This result is explained if we consider that the N-terminus amine function of the proline residue is not essential for PS-binding. To the best of our knowledge, proline residue was not described as a part of the peptide sequence which play a dominant role in the interaction with PS. (Igarashi et al. 1995). However, our peptide has a lower affinity compared to Annexin A5, another imaging agent targeting the PS.

Otherwise, the presence of ^67^Ga-P04087 uptake on autoradiography in contrast to the rapid degradation observed in vitro without the metalloprotease inhibitor, point out the discrepancies that could occur between in vitro and in vivo degradation even when low tracer amounts are used in vitro in order to avoid saturation of enzymes (García-Garayoa et al. 2001). These positive autoradiography results extend the findings of histology experimentations on the binding of the agent to PS. However, comparatively to radiolabelled Annexin A5, the contrast was not sufficient to provide a detectable signal. This lack of signal on the SPECT/CT images could have multiple explanations: 1) ^99m^Tc-AnnexinV has a stronger affinity to PS comparing to Ga-P04087 respectively 6*10^−9^ vs 1.852*10^−7^M. 2) the low intensity of the signal caused by the physical proprieties of ^67^Ga is poorly adapted to the gamma camera. 3) The size of vegetations is small (These vegetations have been imaged successfully with other tracers: ^99m^Tc-AnnexinV with a ratio of 1.3 for in vivo acquisitions and 2 for autoradiography (Rouzet et al. 2008); Annexin A5-128 with similar diagnostic sensitivity than Annexin V (Benali et al. 2014)). 4) ^67^Ga-P04087 have a rapid renal elimination (visualization of a renal excretion on the acquisition image realized 5 min after injection). 5) ^67^Ga-P04087 has lower specific activity than ^99m^Tc-AnnexinV.

Therefore, our work showed that although successful in targeting PS expressed by activated platelets in the rat model of infective endocarditis (autoradiography), the contrast of the signal provided by Ga-P04087 was not sufficient to be detected in vivo.

This lack of signal can reasonably be explained by the lower affinity of ^67^Ga-P04087 compared with Annexin A5-128 (100 fold ratio). To circumvent this drawback, a structure-activity relationship study might be relevant in order to reach for our peptide, a PS affinity comparable to that of Annexin. Reaching high affinity with small peptides remains a realistic challenge (Hofland et al. 2010). Regarding the improvement of the in vivo metabolic stability, many chemical modifications are described and proved to be useful. With this aim and as a first step, we might consider that the design of an analogue peptide of ^67^Ga-P04087 in which l-amino acids would be judiciously replaced by their d-counterparts might retain native peptide affinity but with an improved pharmacological profile (Denèfle et al. 2016).

Although yet unsuccessful, the work realized here is a first step toward the design of a new peptide-based contrast imaging agents targeting PS. Since proteins have several disadvantages over peptides (higher manufacturing costs, higher potential immunogenicity, lower stability, lower activity per mass, worse tissue/organ penetration (Burtea et al. 2009), high liver and kidney uptake (Wuest et al. 2015)), the development of peptide-based radiotracers for imaging apoptosis in vivo remains an interesting and attainable challenge.

## Conclusion

A novel labelled peptide Ga-NODAGA-β-alanine-PGDLSR (Ga-P04087) was synthesized and analysed in vitro and in vivo for its PS-binding properties. Despite the preservation in-vitro of the peptide affinity to PS after chemical modifications and gallium labelling and in spite of the presence of ^67^Ga-P04087 uptake on autoradiography, the absence of detectable foci in vivo makes the peptide used in the present study not suitable as radiotracer for imaging of apoptosis in vivo.

Protein ligands have high affinity to PS but have many drawbacks. Therefore, the development of peptide-based radiotracers for imaging apoptosis in vivo remains a significant challenge. Further studies are necessary to design and evaluate novel radiolabelled peptides with higher binding potencies to PS while possessing enhanced metabolic stability in vivo.

## Abbreviations

DCM: Dichloromethane
DIPEA: Diisopropylethylamine
DMF: Dimethylformamide
FSC: Foetal calf serum
HBTU: O-(Benzotriazol-1-yl)-N,N,N’,N’-tetramethyluronium hexafluorophosphate
HOBt: N-hydroxybenzotriazole
MeOH: Methanol
MS: Mass spectrometry
NMP: N-methyl-2-pyrrolidone
PC: Phosphatidylcholine
PE: Phosphatidylethanolamine
PI: Phosphatidylinositol
PM: Plasma membrane
PS: Phosphatidylserine
SM: Sphingomyelin
TFA: Trifluoroacetic acid
TIS: Triisopropylsilane
